# Rare Anatomic Variation of the Levator Claviculae Muscle

**DOI:** 10.7759/cureus.87022

**Published:** 2025-06-30

**Authors:** Nathaniel G Blanchard, Jenna Farnum, Tristan Packard, Abby L Cummings, William McMillan, Libby J Bradley, Nicole L Geske

**Affiliations:** 1 Anatomy, Michigan State University College of Osteopathic Medicine, East Lansing, USA; 2 Radiology, Michigan State University, East Lansing, USA

**Keywords:** accessory muscle, anatomy, levator claviculae, otolaryngology, prosection

## Abstract

Variations regarding cervical musculature have been well documented in the literature, including the presence of a levator claviculae muscle within the lateral neck. This case report presents an example of levator claviculae that was discovered during the prosection of a 98-year-old male anatomical donor. This accessory muscle was located in the left cervical region and originated from the posterior belly of the levator scapulae, traversed along the anterior border of the trapezius, and inserted onto the inferior clavicle. Branches of the cervical plexus were noted to innervate both the levator scapulae and the levator claviculae. This report reviews these cervical anatomical variations and highlights their clinical significance. By presenting this case, we aim to increase awareness of unusual anatomical variations, which may aid in reducing the potential for misdiagnosis or confusion among healthcare professionals.

## Introduction

The levator claviculae is a cervical accessory muscle found in the posterior triangle of the neck and has a tendency to be unilateral, and when it does occur, it tends to be more frequent on the left side [1]. The muscle is found in roughly 2% to 3% of the population, with many going unnoticed or undocumented during the course of one’s life [2]. Most findings are incidental, found during radiological or cadaveric exams, but intraoperative findings have been discussed in the literature [2]. This muscle has variable descriptions regarding the origin, insertion, and innervation. The consensus across the literature is that it originates from the transverse processes of the C1-C6 vertebrae, with some sources suggesting more cranial origins [1]. The insertion of the muscle occurs along the middle or lateral third of the clavicle, while innervation is commonly provided by spinal nerves in the C2-C6 range [1].

Cervical muscle variation can include more than just the levator claviculae. Current literature suggests that variations in the size, shape, and form of cervical skeletal muscles are relatively common, particularly within the scalene muscle group. Specific findings include significant variation in the “musculotendinous lengths, fascicle lengths, pennation angles, and physiologic cross-sectional areas” of neck muscles involved in head movement. In addition, these prosection studies highlight variations in the tendinous insertion points of the scalene and longissimus capitis muscles between the donors analyzed [3]. 

Regarding the scalene muscles, it has been reported that between 30% and 71% of the population possess a fourth scalene muscle, referred to as the minimum scalene. In addition, individual scalene muscles can exhibit considerable variation in their morphology and insertion/origin location. For example, the anterior scalene typically originates from the anterior tubercles of the C3-C6 vertebrae and inserts onto the first rib; however, it has also been found to originate from C2 and, in some cases, exclude C6 involvement [4].

Variations in the sternocleidomastoid (SCM) muscle have also been documented. For instance, an accessory muscle originating from the hyoid bone and inserting into the fibers of the sternocleidomastoid was discovered during surgery [5]. In a prosection study, an atypical SCM was identified unilaterally on the left side of the donor [6]. This variant consisted of three distinct muscle heads: a medial and lateral clavicular head, along with a sternal head [6]. In addition, an accessory belly of the sternal head merged roughly at the C5 vertebral level with the lateral clavicular head [6].

Overall, anatomical variations are more common than generally recognized, with estimates suggesting that each individual may have up to 10 muscular variants across the body [7]. While the mechanisms behind many of these variations are not yet fully understood, several hypotheses provide logical explanations, including genetic, developmental, and environmental factors [8]. Regardless of underlying causes, it is crucial for clinicians to be aware of these variations, as they have significant implications for diagnostic accuracy. A thorough understanding of these anomalies is particularly important in surgical, radiological, musculoskeletal, and oncological contexts, where failure to recognize such variations could lead to misdiagnosis or unnecessary procedures.

## Case presentation

**Figure 1:**
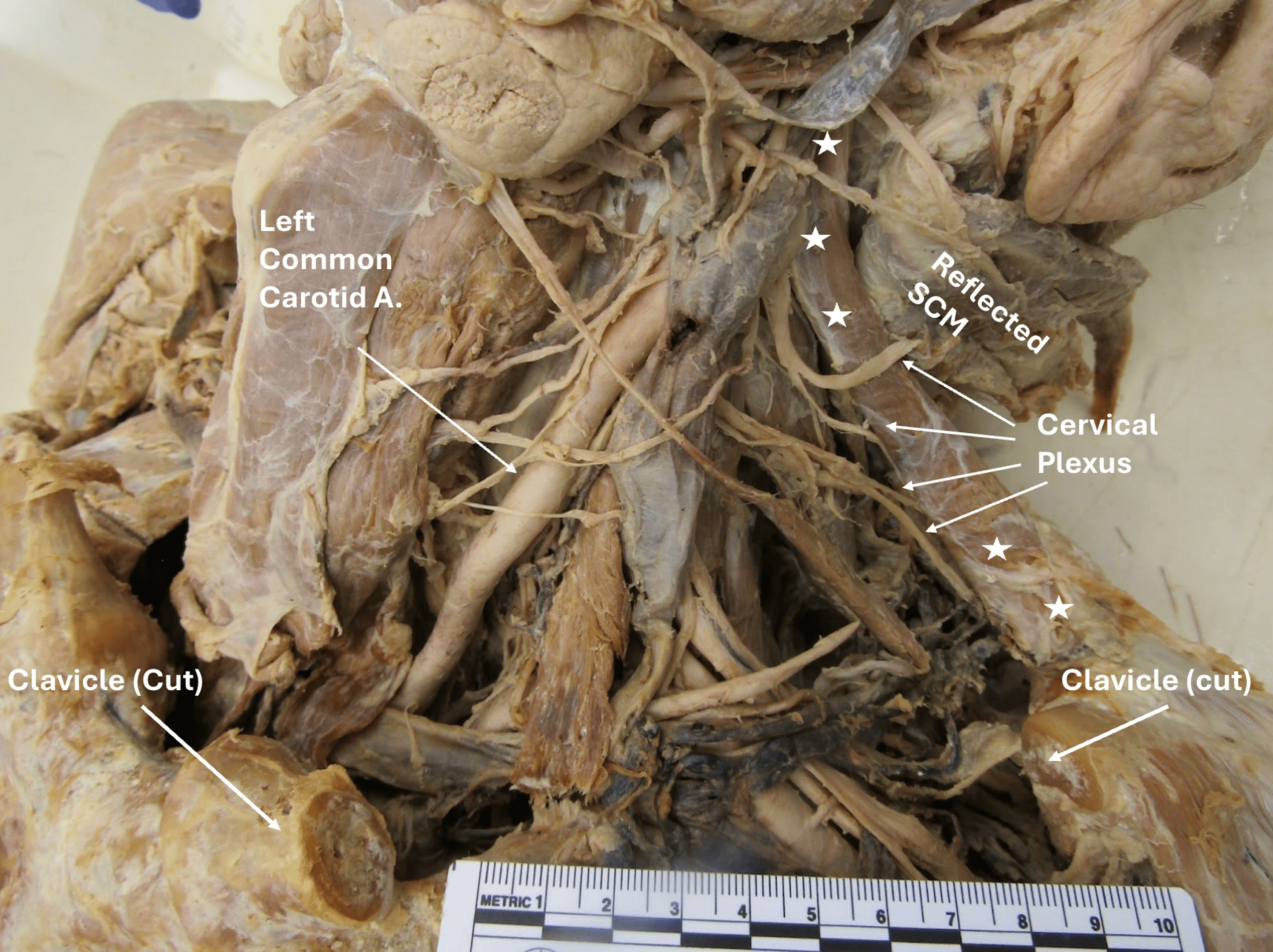
Innervation of left levator claviculae Anterior view of the cervical region highlighting the innervation of levator claviculae by C1-C2. ☆Indicates accessory muscle; SCM: Sternocleidomastoid muscle; Carotid A: Carotid artery

**Figure 2:**
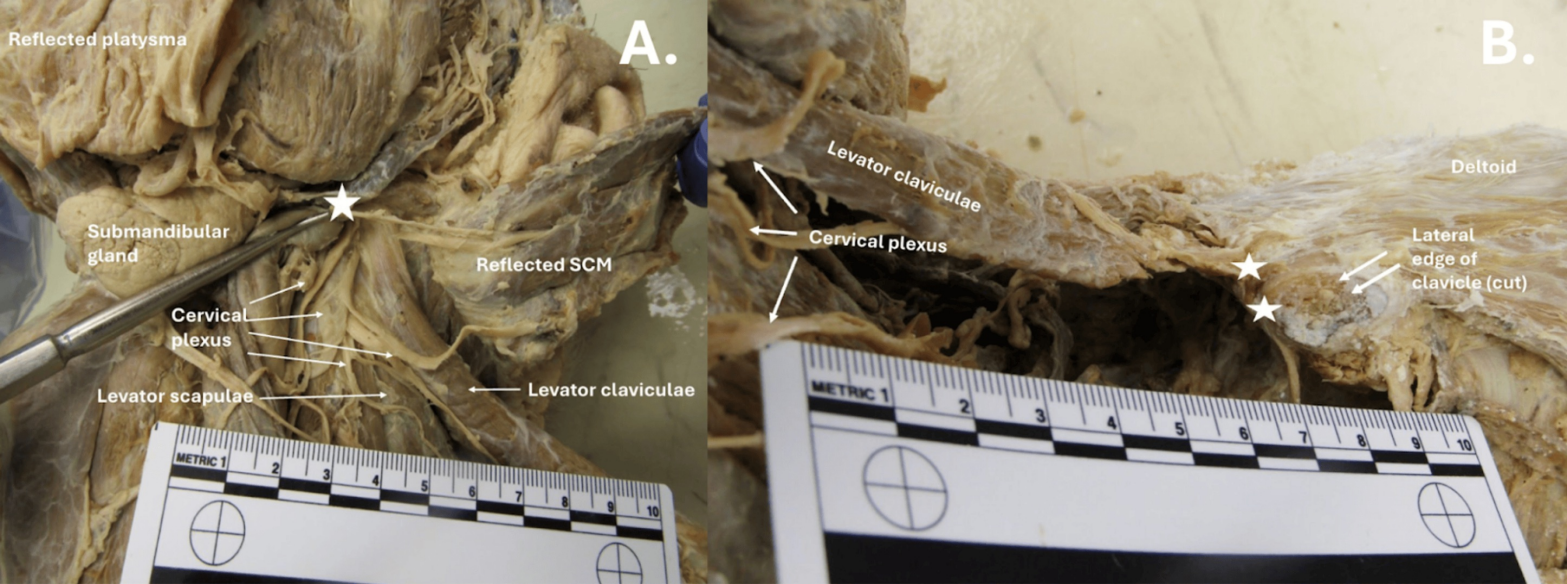
Origin and insertion of levator claviculae Anterior view of the cervical region highlighting the levator claviculae originating from the levator scapulae on the transverse process of C1 and inserting on the inferolateral aspect of the clavicle. A: ☆ indicates the origin of levator claviculae; B: ☆ indicates the inferior insertion of levator claviculae SCM: Sternocleidomastoid muscle

During a routine prosection of a 98-year-old male anatomical donor, an anatomical variation was identified in the left cervical region (Figure 1). An accessory muscle,the levator claviculae, was observed originating from the cervical transverse process of C1 in continuity with the levator scapulae muscle (Figure 2). This levator claviculae muscle was distinct from the sternocleidomastoid and inserted on the inferior aspect of the most lateral third of the clavicle (Figure 2). Notably, the muscle was innervated by the C1-C2 branches of the cervical plexus (Figure 1).

## Discussion

**Figure 3:**
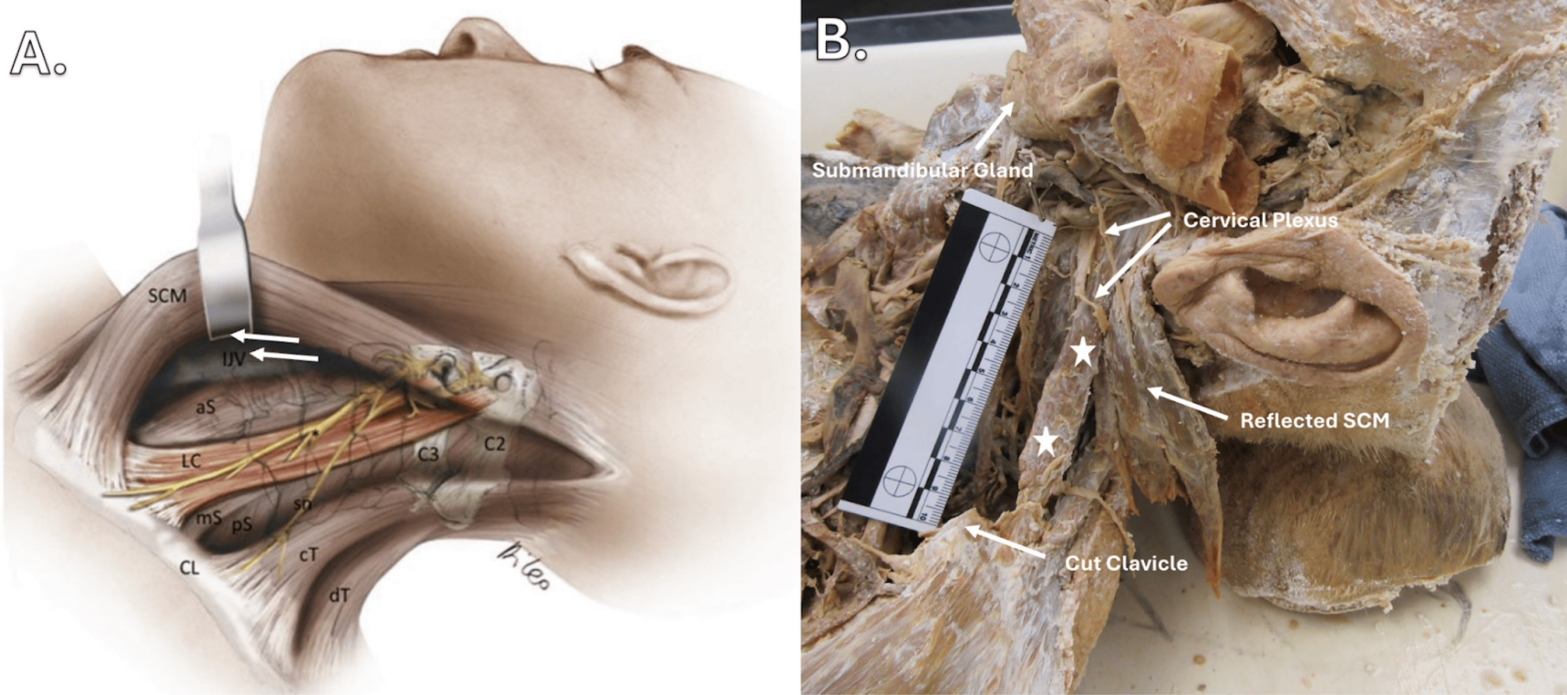
Lateral view of the cervical region The illustration of the levator claviculae and surrounding cervical structures in panel A has been reused with the permission of John Wiley & Sons Ltd. from the article 'Levator Claviculae Muscle: Anatomic Variation Found During Neck Dissection Variation of the Levator Claviculae Muscle' by Ferreli et al. [2]. Panel B shows how the levator claviculae merges with levator scapulae before originating on the transverse process of C1. It also shows the distal insertion of levator claviculae on the inferolateral surface of the clavicle. ☆Indicates the orientation of the levator claviculae’s muscle body in panel B. SCM: Sternocleidomastoid muscle; IJV: Internal jugular vein; aS: Anterior scalene muscle; LC: Levator claviculae; mS: Middle scalene muscle; pS: Posterior scalene; sn: Spinal nerve; C3: c3 vertebrae; C2: C2 vertebrae; CL: Clavicle; cT and dT: Cervical and dorsal parts of the trapezius

As illustrated in Figure 3, the accessory muscle of the anatomical donor (panel B) and the artistic rendering of the levator claviculae (panel A) exhibit striking similarities in muscle morphology and their association with adjacent cervical structures. As discussed previously, the levator claviculae muscle typically originates from the transverse processes of the C1-C6 vertebrae and inserts on the middle or lateral third of the clavicle [9]. However, a notable difference is observed in this case, in which the origin of the muscle appears to blend with the proximal aspect of the levator scapulae muscle before connecting to the transverse process of C1. In addition, the muscle described in this case inserts on the inferolateral surface of the clavicle instead of the medial or lateral third of the clavicle [1]. These findings suggest that the described variant may have unique origins and insertion locations. In a similar pattern, the levator claviculae of the donor presented in this case appears to receive innervation from higher than normal spinal nerves, including C1 and C2, instead of C2-C6, which is more commonly referenced across the literature [1]. 

Although discrepancies in the description of the levator claviculae's origin, insertion, and innervation exist across the literature, consistent evidence supports its overall relationship with the cervical spine, cervical plexus, and clavicle. These discrepancies likely reflect natural variation in the accessory muscle, which explains why the muscle in this case study exhibits slight deviations from the typical anatomical characteristics of the levator claviculae.

The embryonic development of the levator claviculae remains poorly understood, but several hypotheses offer plausible explanations for its formation. These include the muscle's potential origin from the sternocleidomastoid, longus colli, trapezius, anterior scalene, or an extra segment of the ventrolateral muscle primordia of the neck [10]. Another hypothesis suggests contributions from the levator scapulae [11]. Given the shared innervation (C1-C2) of several neck muscles, including the ventral vertebral muscles, hyoid muscles, scalenes, and sternocleidomastoid, the authors also consider the possibility that the described variant originates from a similar source within the primaxial domain of the embryonic mesoderm. Based on innervation, this hypothesis is supported by previous reports of levator claviculae occurrence [1].

Overall, there are numerous points in the developmental process at which environmental and genetic factors may influence muscle structure. While the exact mechanism for the variation described in this case is unclear, the authors lean towards the suggestion of a genetic component in the development of this accessory muscle, given its similarities to the levator claviculae. Moreover, the muscle of interest shares its fibers with the levator scapulae in its upper portion and therefore likely shares the same origin.

Clinical significance 

The presence of an accessory muscle, such as the one described in this case study, and the variant known as the levator claviculae, has clinical relevance, particularly with thoracic outlet syndrome (TOS). This syndrome results from compression of the neurovascular structures in the cervicoaxillary region and can be exacerbated by anatomical variations such as an accessory muscle in the region [12]. The levator claviculae, often presenting unilaterally, has been incidentally identified in radiographic findings of patients diagnosed with TOS [12]. This anatomical variant may contribute to the development of TOS through its proposed function of elevating the rib cage or assisting in neck rotation, actions that could lead to compression of the underlying neurovascular structures, thereby implicating the muscle in the pathophysiology of TOS [9]. Recognizing the musculoskeletal origin of compression can alter the prognosis and guide treatment options, including osteopathic manipulative medicine (OMM). The OMM techniques, such as targeted stretching, muscle strengthening, and icing, have been shown to provide significant symptom relief and pain reduction in some patients within a relatively short period [12].

Awareness of anatomical variations is also crucial during head and neck surgeries. Surgeons rely on well-established anatomical landmarks and patterns to guide their decisions [5]. Variations in anatomy, such as the presence of an accessory muscle like the levator claviculae, can complicate the identification of structures during surgery and lead to misidentification, increasing the risk of adverse outcomes [5]. While the levator claviculae is not a common variant-occurring in only approximately 2% to 3% of the population, surgeons need to be aware of its potential presence, particularly during complex neck dissections [2]. Without this awareness, surgical teams could misinterpret the variant as a mass, such as a tumor or lymph node, leading to unnecessary diagnostic procedures or interventions.

Similarly, but in the context of radiology, the presence of an accessory muscle like the levator claviculae can be mistaken for a pathological mass in the cervical region [13]. This misinterpretation can lead to unnecessary medical workup and delays in the accurate diagnosis. Therefore, understanding this anatomical variation is vital for radiologists and surgeons to avoid confusion and to ensure appropriate treatment planning [5].

In summary, recognizing and understanding anatomical variations, such as the levator claviculae, can enhance more targeted clinical care, prevent misdiagnosis, and ultimately improve patient outcomes. By raising awareness of these variations, medical professionals are better equipped to provide personalized help to patients and make informed decisions about the care they receive.

## Conclusions

During the routine prosection of a 98-year-old male anatomical donor, an unusual unilateral accessory muscle was discovered in the left cervical region. This muscle originated from the transverse process of C1, with fibers blending with the levator scapulae and inserted to the inferior surface of the lateral-most third of the left clavicle. Cervical anatomical variations are relatively common and can present challenges in clinical and surgical settings. This case study aims to summarize existing findings and contribute to the growing body of literature on cervical muscle variations by offering a specific example, namely the levator claviculae, found in the donor. By highlighting this rare anatomical presentation, the authors aim to raise awareness among medical professionals, including surgeons, radiologists, and physicians, regarding the potential implications of accessory muscles in clinical practice. Such knowledge is critical for accurate diagnosis, management, and surgical planning.
